# Comment on “An Analysis of 3-Year Outcomes following Canaloplasty for the Treatment of Open-Angle Glaucoma”

**DOI:** 10.1155/2018/4257512

**Published:** 2018-09-27

**Authors:** Afaf Abdullah Bin Khathlan

**Affiliations:** Al Hayat Medical Group, Riyadh, Saudi Arabia

With interest, I have read the observational study by Khaimi et al. on the three-year outcomes of canaloplasty [1]. I would like to point out some methodological issues in the statistical analysis that hindered the complete assessment of the generalizability of the study's results.

The STrengthening the Reporting of OBservational studies in Epidemiology (STROBE) statement published a checklist for items to be addressed in observational studies, which was not followed in this study [2]. Here are some concerns observed:

1. Complications: Visual acuity was statistically lower after surgery until 1-month follow-up for all eyes (Table 2) and until 6 months in canaloplasty only (Table 3). This drop in vision was not fully explained nor analyzed by the authors. Intraoperative complications were not fully reported as well. Other than hyphema, the study failed to report other complications.
2. Using mean for ordinal data: The authors reported the use of medications as mean and SD, but it is easier to interpret ranked data using median with interquartile range. Results will need to be presented in bar charts with percentage of cases on single, 2, or 3 medications postoperatively on first and final follow-up in place of a mean Meds 0.6 (SD 0.9).
3. Missing data: In Tables 2 and 3 and to lesser extent in Table 4, the number of cases analyzed was different for the three outcome measures (mean IOP, Meds, and visual acuity). It is highly recommended to drop those cases with missing outcomes from the analysis for each visit if they are within the 5% rate. As an example, in Table 2 at 18 months' visit, there were 167 cases in the IOP outcome, 144 cases in the number of Meds outcomes, and 172 cases in the visual outcome, but it is not clear if the same case was included for all three outcomes and how the 32 cases with vision-only outcome and who had missing Meds information were interpreted.
4. Attrition rate: The attrition rate was 35% at the 12-month follow-up and up to 76% at the three-year follow-up. Further analysis using the *t*-test for the differences between the two groups (retained and lost to follow-up) is necessary to avoid potential bias [3].
5. Exclusion criteria: It was noticed in the Discussion that cases with failed suture tension placement were excluded from analysis in this study. This was not clearly outlined in patients' selection criteria of the patients and methods or the results sections.
6. Subgroup analysis: The cases included were POA and pigmentary and exfoliation glaucoma. There was no subanalysis for each group. The authors could not state in Conclusions that “canaloplasty's safety profile and long-term efficacy make it a viable option for the majority of glaucoma patient types” without the subanalysis to support this statement.

It is important to highlight that observational studies are extremely important, specifically long-term outcomes, to report benefits and harm of surgical and medical interventions.

Finally, I would like to thank the authors for their efforts, but I would like to stress that the results from this study should be interpreted with caution due to the statistical and methodological shortcomings observed.
